# Urban–rural and gender differences in tobacco and alcohol use, diet and physical activity among young black South Africans between 1998 and 2003

**DOI:** 10.3402/gha.v6i0.19216

**Published:** 2013-01-29

**Authors:** Nasheeta Peer, Debbie Bradshaw, Ria Laubscher, Nelia Steyn, Krisela Steyn

**Affiliations:** 1 Chronic Diseases of Lifestyle Research Unit, Medical Research Council, Durban, South Africa; 2 Burden of Disease Research Unit, Medical Research Council, Cape Town, South Africa; 3 Biostatistics Unit, Medical Research Council, Cape Town, South Africa; 4 Population Health, Health Systems and Innovation, Human Sciences Research Council, Cape Town, South Africa; 5 Department of Medicine, University of Cape Town, Cape Town, South Africa

**Keywords:** South Africa, young adults, lifestyle risk factors, smoking, alcohol, obesity, physical activity

## Abstract

**Background:**

Non-communicable chronic diseases (NCDs) have increased in South Africa over the past 15 years. While these usually manifest during mid-to-late adulthood, the development of modifiable risk factors that contribute to NCDs are usually adopted early in life.

**Objective:**

To describe the urban–rural and gender patterns of NCD risk factors in black adolescents and young adults (15- to 24-year-olds) from two South African Demographic and Health Surveys conducted 5 years apart.

**Design:**

An observational study based on interviews and measurements from two cross-sectional national household surveys. Changes in tobacco and alcohol use, dietary intake, physical inactivity, and overweight/ obesity among 15- to 24-year-olds as well as urban–rural and gender differences were analysed using logistic regression. The ‘Surveyset’ option in Stata statistical software was used to allow for the sampling weight in the analysis.

**Results:**

Data from 3,186 and 2,066 black 15- to 24-year-old participants in 1998 and 2003, respectively, were analysed. In males, the prevalence of smoking (1998: 21.6%, 2003: 19.1%) and problem drinking (1998: 17.2%, 2003: 15.2%) were high and increased with age, but in females were much lower (smoking – 1998: 1.0%, 2003: 2.1%; problem drinking – 1998: 4.2%, 2003: 5.8%). The predominant risk factors in females were overweight/obesity (1998: 29.9%, 2003: 31.1%) and physical inactivity (2003: 46%). Urban youth, compared to their rural counterparts, were more likely to smoke (odds ratio (OR): 1.39, 95% confidence interval (CI): 1.09–1.75), have high salt intake (OR: 1.75, 95% CI: 1.12–2.78), be overweight/obese (OR: 1.39, 95% CI: 1.14–1.69), or be physically inactive (OR: 1.45, 95% CI: 1.12–1.89). However, they had lower odds of inadequate micronutrient intake (OR: 0.46, 95% CI 0.34–0.62), and there was no overall significant urban– rural difference in the odds for problem drinking but among females the odds were higher in urban compared to rural females.

**Conclusion:**

Considering that the prevalence of modifiable NCD risk factors was high in this population, and that these may persist into adulthood, innovative measures are required to prevent the uptake of unhealthy behaviours, and regular surveillance is needed.

Chronic non-communicable diseases (NCDs), such as cardiovascular disease, type 2 diabetes, cancer, and chronic obstructive pulmonary disease, have increased in South Africa over the past 15 years (1). These are predicted to rise considerably over the next decades if measures are not introduced to curb the NCD burden (1). The World Health Organization (WHO) estimated that in 2004, NCDs were responsible for 28% of the disease burden measured by disability-adjusted life years (DALYs) in all South Africans (2).

While NCDs usually manifest during mid-to-late adulthood, modifiable risk factors that contribute to these conditions are generally adopted early in life (3). For example, smoking onset, regular use, and dependence among the majority of adult smokers usually begins during adolescence (ages 10–18 years) (4), and initiation of alcohol use during the pre- and early adolescent years increases the vulnerability to alcohol use disorders in later life (5). Furthermore, the ‘obesogenic’ environment seems to be mainly directed at the adolescent market, thereby making healthy choices more difficult (6). The ‘obesogenic’ environment encourages unhealthy dietary habits and decreased physical activity through indoor activities such as television viewing during adolescence (6).

Behavioural (smoking, alcohol abuse, physical inactivity, and poor dietary habits) and physiological (obesity) NCD risk factors are known to be prevalent in the South African youth (7). However, less is known about the pattern of these risk factors during the transition into adulthood and how this relates to urban or rural settings in the country. South Africa is a middle income country with large wealth disparities and increasing proportions of the population living in urban areas (8). Understanding the differences in the pattern of, and the factors associated with, the NCD risk factors among the youth will contribute to NCD prevention planning and health policy and research programmes (9). Analysis of the secondary data for 15- to 24-year-olds from two cross-sectional surveys was undertaken to assess changes over time and examine the differences between urban and rural settings, and male and female genders.

## Methodology

The population-based South African Demographic and Health Surveys (SADHS) conducted in 1998 and 2003 collected data, including an adult health module for those ≥15 years of age, from a nationally representative, multistage cluster sample of households. The same methodology, outlined in the respective reports (10, 11), was used to assess both sample populations. Structured questionnaires (translated into the 11 official languages) and anthropometric measures were administered by trained fieldworkers.

Risk factors analysed included self-reported tobacco and alcohol use, physical inactivity, poor dietary intake, and measures of overweight/obesity. The 1998 SADHS adult health questionnaire utilised the WHO step-wise surveillance programme questions (12) and the CAGE set of four questions (Have you ever felt that you should cut down on your drinking? Have people annoyed you by criticizing your drinking? Have you ever felt bad or guilty about your drinking? Have you ever had a drink first thing in the morning to steady your nerves or get rid of a hangover?) to assess problem drinking (13). The 1998 survey instrument was expanded in 2003 to include nutrition and physical activity data. A validated nutrition assessment based on a brief 30-item food frequency questionnaire designed for micronutrient intake (10), and questions on fat and salt intake as well as physical activity questions based on the Global Physical Activity Questionnaire (GPAQ) (14) were obtained. Prior to the survey, the instrument had been tested on 215 South Africans and found to be reliable with criterion validity (10). It has also been validated in other developing country settings (15). Both surveys measured anthropometry using standardised techniques involving standard equipment, uniform training, and supervision and calibration of scales.

Problematic alcohol use was deemed positive if ≥2 CAGE questions were answered affirmatively (13). The adjusted metabolic equivalent level (METS/week) of self-reported physical activity was calculated from the GPAQ responses. The amount of exercise providing cardiovascular benefit was defined as ≥600 METS/week, and levels below this were considered as physical inactivity (14). A micronutrient score was calculated from the 30-item food frequency questionnaire developed for South Africa by estimating the intake of 13 nutrients (10). A score for each nutrient, based on the proportion of the recommended daily allowance, was totalled, with a higher score indicative of poorer micronutrient consumption (10). A cut-off value of 36+, based on the top quintile and equivalent to ≥80% of the total scores in South African adults, defined inadequate micronutrient intake. Fat and salt intakes were similarly determined using scoring systems with 3.5+ and 6+, respectively, equivalent to ≥80%, defining high intake. Body mass index (BMI), calculated as weight in kilograms divided by height in metres squared (kg/m^2^), categorised normal weight (18.5–24.99), overweight (25–29.99), and obesity (≥30) (16). Raised waist-to-hip ratio (WHR), an indicator of fat distribution, was calculated using waist divided by hip measurement, and recommended cut-offs of >1.0 in males and >0.85 in females were used (16).

Urban and rural areas were based on the categorisation used by Statistics South Africa with urban including formal and informal areas and rural including farming and traditional areas (17).

The analysis was restricted to the black population who comprise the majority of the population and are the only group with adequate numbers living in both the urban and rural settings. The data were weighted to account for the sample design and the response rate in each survey so as to provide nationally representative estimates for each year. Logistic regression was used to investigate the association between the NCD risk factors and gender, age, residence, and time period. The odds ratio (OR) and 95% confidence interval (95% CI) for each NCD risk factor was calculated for each characteristic (unadjusted). For the multivariate analysis, the most parsimonious model was selected based on the significance of effects (*P*<0.05), starting with a model with the highest order interaction term and removing effects that were not statistically significant. The adjusted OR and 95% CI were calculated for each characteristic and significant interaction term (adjusted), and the maximum rescaled *R*^2^ was used to assess the goodness of fit.

The Ethical Committee of the South African Medical Research Council approved the SADHS study protocols. The National Department of Health granted permission for the analysis of the SADHS data reported here.

## Results

The response rate in 1998 was 76% for males and 91% for females, and in 2003, it was 79% and 84% for males and females, respectively. Males comprised 47% of this realised sample in both surveys. Similar to the other age groups, the proportion of 15- to 24-year-olds living in urban areas increased from 55% in 1998 to 63% in 2003. The black African population comprised 82% of the youth sample in 1998 and 87% in 2003. There were 3,186 and 2,066 black participants aged 15–24 years in the 1998 and 2003 surveys, respectively, representing 0.05% and 0.03% of the black African population in this age group in 1998 and 2003, respectively.

**Fig. 1:**
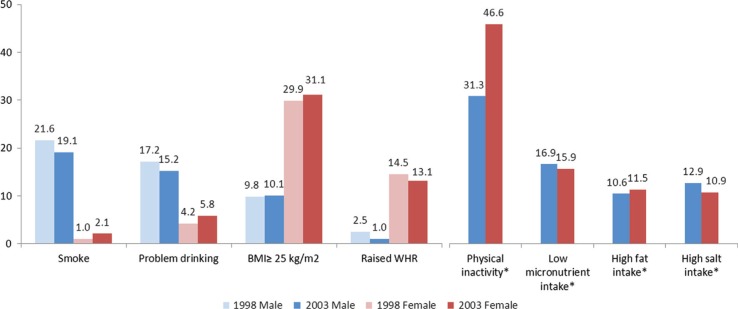
Prevalence of NCD risk factors in black 15- to 24-year-old males and females in 1998 and 2003. *Data available for 2003 only.

Figure 1 describes the NCD risk factors in 15- to 24-year-old black males and females in 1998 and 2003 presented by sex and year/period. Males, compared to females, had a higher prevalence of smoking (1998: 21.6% vs. 1.0%; 2003: 19.1% vs. 2.1%) and problem drinking (1998: 17.2% vs. 4.2%; 2003: 15.2% vs. 5.8%). These prevalences increased with age in both males and females, with the increase being particularly marked for males (data provided in Appendix Table 1).

**Table 1:** Unadjusted and adjusted odds ratios for selected non-communicable chronic disease risk factors in 15- to 24-year-old black youth

	Smoke daily/occasionally				Problem drinking: CAGE ≥ 2				BMI ≥25 kg/m^2^				Raised waist-to-hip ratio			
			
	Unadjusted		Adjusted		Unadjusted		Adjusted		Unadjusted		Adjusted		Unadjusted		Adjusted	
							
	OR	95% CI	OR	95% CI	OR	95% CI	OR	95% CI	OR	95% CI	OR	95% CI	OR	95% CI	OR	95% CI
Age (years)																
15–16	1.00	–	1.00	–	1.00	–	1.00	–	1.00	–	1.00	–	1.00	–	1.00	–
17–18	3.52	2.13–5.81	3.69	2.23–6.11	1.90	1.32–2.75	1.91	1.30–2.79	1.13	0.86–1.49	1.14	0.86–1.51	1.22	0.87–1.72	1.24	0.87–1.77
19–20	5.56	3.43–9.03	6.77	4.08–1.24	2.88	2.03–4.08	3.16	2.19–4.55	1.81	1.37–2.39	1.77	1.33–2.35	1.15	0.83–1.59	1.08	0.77–1.50
21–22	7.24	4.55–1.51	9.93	6.08–6.22	3.35	2.31–4.86	3.84	2.60–5.68	2.54	1.92–3.36	2.35	1.75–3.17	1.21	0.85–1.72	1.12	0.78–1.59
23–24	8.06	4.92–13.2	11.86	7.06–9.94	4.27	2.85–6.39	5.16	3.41–7.8	2.86	2.17–3.77	2.58	1.94–3.43	1.68	1.18–2.40	1.53	1.06–2.21
Sex																
Male	1.00	–	1.00	–	1.00	–	1.00	–	1.00	–	1.00	–	1.00	–	1.00	–
Female	0.06	0.04–0.09	0.03	0.02–0.05	0.26	0.20–0.33	0.28	0.20–0.41	3.96	3.25–4.84	3.90	3.19–4.77	5.37	3.95–7.29	5.33	3.93–7.25
Residence																
Rural	1.00	–	1.00	–	1.00	–	1.00	–	1.00	–	1.00	–	1.00	–	1.00	–
Urban	1.45	1.18–1.82	1.39	1.09–1.75	1.22	0.97–1.52	0.95	0.74–1.23	1.45	1.22–1.72	1.39	1.14–1.69	0.92	0.70–1.20	0.87	0.67–1.14
Period																
1998	1.00	–	1.00	–	1.00	–	1.00	–	1.00	–	1.00	–	1.00	–	1.00	–
2003	0.96	0.75–1.22	0.82	0.62–1.09	1.00	0.79–1.27	0.99	0.77–1.27	1.03	0.86–1.24	1.09	0.88–1.34	1.19	0.90–1.59	1.24	0.94–1.64
Interactions																
Period×residence x sex											1.32	1.08–1.61				
Residence×sex							1.75	1.08–2.86			0.97	0.80–1.19				
Period×sex			2.76	1.11–6.83							0.96	0.79–1.18				
Period×residence											1.04	0.85 1.27				
Maximum-rescaled R^2^				0.3081				0.1261				0.1440				0.1020

	High salt intake*				High fat intake*				Low micronutrient intake*				Physical activity <600 METS/week*			
			
	Unadjusted		Adjusted		Unadjusted		Adjusted		Unadjusted		Adjusted		Unadjusted		Adjusted	
							
	OR	95% CI	OR	95% CI	OR	95% CI	OR	95% CI	OR	95% CI	OR	95% CI	OR	95% CI	OR	95% CI

Age (years)																
15–16	1.00	–	1.00	–	1.00	–	1.00	–	1.00	–	1.00	–	1.00	–	1.00	–
17–18	1.31	0.80–2.14	1.32	0.8–2.18	0.89	0.56–1.41	0.88	0.55–1.41	0.92	0.65–1.32	0.93	0.65–1.33	1.12	0.80–1.55	1.09	0.77–1.54
19–20	1.17	0.66–2.09	1.18	0.66–2.11	1.25	0.75–2.08	1.25	0.75–2.08	0.83	0.56–1.22	0.83	0.56–1.22	0.93	0.63–1.36	0.90	0.61–1.31
21–22	1.04	0.58–1.87	1.02	0.56–1.84	1.09	0.61–1.94	1.06	0.59–1.89	0.79	0.52–1.21	0.84	0.56–1.28	1.68	1.16–2.43	1.62	1.10–2.37
23–24	1.19	0.66–2.12	1.15	0.64–2.07	1.42	0.80–2.55	1.38	0.77–2.45	0.61	0.39–0.96	0.66	0.42–1.03	1.73	1.17–2.57	1.59	1.05–2.40
Sex																
Male	1.00	–	1.00	–	1.00	–	1.00	–	1.00	–	1.00	–	1.00	–	1.00	–
Female	0.83	0.56–1.23	0.83	0.55–1.23	1.09	0.76–1.57	1.08	0.75–1.55	0.93	0.71–1.21	0.94	0.72–1.23	1.84	1.43–2.36	1.83	1.42–2.35
Residence																
Rural	1.00	–	1.00	–	1.00	–	1.00	–	1.00	–	1.00	–	1.00	–	1.00	–
Urban	1.75	1.12–2.70	1.75	1.12–2.78	1.33	0.94–1.89	1.32	0.93–1.89	0.45	0.34–0.61	0.46	0.34–0.62	1.49	1.16–1.92	1.45	1.12–1.89
Maximum-rescaled *R* ^2^				0.0185				0.0093				0.0402				0.0605

* Data available for 2003 only.

Low levels of physical activity, obtained in 2003 but not in 1998, were higher in females (46.6%) compared to males (31.3%). Poor nutrition, determined by low micronutrient intake and high fat and salt intake, ascertained only in 2003, was similar in males and females. Low micronutrient intake, at 16.9% in males and 15.9% in females, was the most prevalent of the poor nutrition indicators.

The prevalence of overweight/obesity was similar in 1998 and 2003 with higher prevalence in females (1998: 29.9%; 2003: 31.1%) compared to males (1998: 9.8%; 2003: 10.1%). Raised WHR was also higher in females compared to males in 1998 (14.5% vs. 2.5%) and 2003 (13.1% vs. 1.0%).

**Fig. 2:**
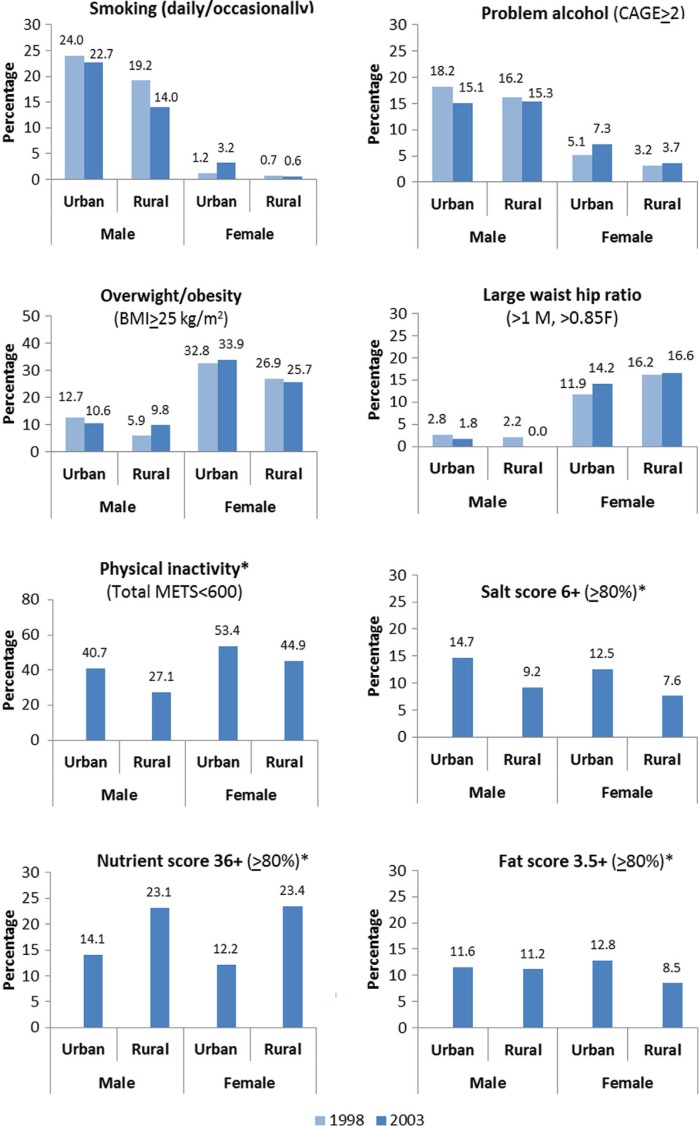
Prevalence of behavioural and physiological risk factors among African youth by residence and sex, 1998 and 2003. *Data available for 2003 only.

The prevalence of risk factors among 15- to 24-year-old black males and females presented by urban–rural residence is illustrated in Fig. 2. When comparing the risk factors in 2003, physical inactivity was the predominant contributor to NCD risk, ranging from 27.1% in rural males to 53.4% in urban females. Among rural males, inadequate micronutrient intake was a major contributor to NCD risk, ranking second at 23.1%, ahead of problem drinking (15.3%) and smoking (14.0%). Inadequate micronutrient intake in rural females, at 23.4%, ranked third, after physical inactivity (44.9%) and overweight/obesity (25.7%). Of the NCD risk factors among urban males in 2003, physical inactivity (40.7%), smoking (22.7%), and problem drinking (15.1%) predominated. Among urban females, a high level of physical inactivity in 2003 was accompanied by a high prevalence of overweight/obesity (33.9%).

Table 1 reports the unadjusted and adjusted ORs for the relationship of each risk factor with age, gender, urban–rural, and time period. Regarding time, there was no significant change in the unadjusted odds for risk factors measured in both 1998 and 2003. However, a significant period–residence–sex interaction was observed in the odds of overweight/obesity in the multivariate model. The odds of overweight/obesity in urban females in 2003 was significantly higher than the odds of rural males in 1998 (OR: 1.32, 95% CI: 1.08–1.61), after adjusting for the increase with age. From Fig. 2, it can be seen that the prevalence of overweight/obesity increased in rural males (5.9% vs. 9.8%) and in urban females (32.8% vs. 33.9%), while the prevalence decreased in urban males (12.7% vs. 10.6%) and rural females (26.7% vs. 25.7%). Females compared to males had significantly higher adjusted odds for overweight/obesity (OR: 3.90, 95% CI: 3.19–4.77), raised WHR (OR: 5.33, 95% CI: 3.93–7.25), and physical inactivity (OR: 1.83, 95% CI: 1.42–2.35). The odds for being overweight/obese increased in those aged 19 years and above.

There was a significant interaction effect between time period and sex for the odds of smoking (OR: 2.76, 95% CI: 1.11–6.83). This reflects the decrease in the prevalence of smoking for males from 1998 to 2003 (21.6% vs. 19.1%) and the slight increase in females for the same period (1.0% vs. 2.0%). The difference in the odds of smoking between males and females was extremely marked when adjusted for the other factors (OR: 0.03, 95% CI: 0.02–0.05). There was a significant urban–rural and gender interaction effect in the odds of problem drinking (OR: 1.75, 95% CI: 1.08–2.86). From Fig. 1, it can be seen that the prevalence of problem drinking decreased in males (17.2% vs. 15.2%), whereas it increased in females (4.2% vs. 5.2%). Of note is the lack of difference in the adjusted odds of problem drinking between rural and urban areas (OR: 0.95, 95% CI: 0.74–1.23).

There were no significant gender differences in dietary intake in 2003 as shown in Table 1. The comparison by residence demonstrated that urban, compared to rural residents, had significantly higher salt intake (OR: 1.75, 95% CI: 1.12–2.78), but a lower adjusted odds for inadequate micronutrient intake (OR: 0.46, 95% CI: 0.34–0.62) in 2003. Of note is that problem drinking was pervasive in both areas (OR: 1.05, 95% CI: 0.81–1.36). There was no significant urban–rural difference in the adjusted odds of high fat intake (OR: 0.76, 95% CI: 0.53–1.07). It is noticeable that the dietary indicators ascertained in 2003 did not change significantly with age between 15 and 24 years.

## Discussion

The prevalence of NCD risk factors among black adolescent and young adult South Africans from 1998 to 2003 has remained high and essentially unchanged. The lack of a decline in the prevalence of NCD risk factors is not surprising, since there have been few concerted interventions to address these unhealthy lifestyles (18).

The only public health measure of note has been the introduction of tobacco control policies in South Africa in 1993 (19), which resulted in a decline in smoking in ≥15-year-old men, from 42% in 1998 to 35% in 2003 (10, 11). However, of concern is the fact that although there has been a decline in the smoking prevalence in young males, the prevalence remains high, particularly in the urban youth. Moreover, the use of tobacco products among 13- to 15-year-old South African students was higher than that of most other countries studied (20). This highlights the minimal impact that the tobacco control policies have had on the South African youth. Of note is the fact that awareness of the dangers of smoking in the youth are probably minimal as only 38.7% of 13- to 15-year-old South African scholars reported having being taught about the dangers of smoking in school (20). The recent amendment to the tobacco control policy, which has increased the minimum age of sales from 16 to 18 years, may assist in delaying cigarette uptake (21). However, the determinants of smoking initiation in young South Africans need to be studied and addressed.

Moreover, while much lower compared to males on account of social norms, the increasing smoking uptake among young females in urban areas is of particular concern. The social and cultural constraints that previously prevented black females from smoking are weakening (22, 23), especially in urban areas, and traditional constraints can no longer be relied on to maintain the relative low smoking prevalence (23). This study highlights that late adolescence is a critical age for the uptake of tobacco smoking.

Unlike the significant urban–rural difference for smoking, the prevalence of problem drinking was similarly high in both rural and urban male residents, demonstrating the pervasive distribution of alcohol in South Africa. Tsering and colleagues also reported no difference in alcohol use among urban and rural scholars, mean age 15 years, in India (24). The high and sustained prevalence of problem drinking in 15- to 24-year-old males is a manifestation of the prevailing societal norms and attitudes that favour erratic heavy drinking (25). Moreover, on account of the relatively low price of alcohol in South Africa, access to alcoholic beverages is easy and may have contributed to easier uptake and over-indulgence among the youth (25). Despite the introduction of legislation in South Africa, The Liquor Act of 2003, aimed at reducing the socio-economic effects of alcohol abuse and protecting children by prohibiting advertising to minors (18), the control of alcohol use through legislation has had little impact on problematic alcohol use (1).

Also of concern is the fact that the age of initiation of alcohol use among South African youth has lowered over the years (7). Initiatives that reduce youth drinking are urgently required, as heavy consumption in adolescence or young adulthood often precedes alcohol abuse later in life (26). Increased alcohol taxation may be of potential benefit as young drinkers are likely to be responsive to price. A study among Brazilian adolescents found a higher frequency of alcohol consumption among those with, compared to without, personal income (27) highlighting the relationship between alcohol consumption and affordability.

A review of dietary trends in South Africa based on multiple data sources suggests that the nutrition transition, from a traditional diet high in carbohydrate and fibre and low in fat to a diet higher in fat and salt, is evident and associated with urbanisation trends (8). This study confirmed the association of higher salt intake with urbanisation but showed no significant difference in fat intake between rural and urban residents. The lack of an overall observed difference in fat intake may be because the dietary measure used in the SADHS had limited questions on fat consumption since it is a screening tool with a focus on micronutrient intake. However, an anthropometric study in a rural setting by Kimani-Murage et al. (28) observed both under-nutrition and a substantial prevalence of overweight and obesity among adolescent girls. The authors suggest that insufficient local food production in rural areas may have led to dependence on energy-dense processed foods and marked changes in traditional diets (28).

However, females residing in urban areas had a higher prevalence of high fat intake compared to their rural counterparts (12.8% vs. 8.5%). The study suggests that with further urbanisation, the prevalence of high fat intake may increase in future.

The higher prevalence of inadequate micronutrient intake in rural compared to urban populations reported in this study has been noted previously, and interpreted to be the outcome of lower income and a poorer quality diet which has little diversity (8). A Brazilian study found that males, younger adults, lower education, poorer households, and rural residence were associated with low fruit and vegetable intake, which are primary sources of micronutrients (29). The high prevalence of inadequate micronutrient intake in rural South Africans should be noted and probably warrants further investigation. The government's recommendation to eat ‘plenty of fruit and vegetables every day’ (30) needs to be actively promoted with these foods made easily accessible and affordable to the rural poor.

The high levels of inactivity in these youth are of concern and can only be expected to worsen as longitudinal studies report a decrease in physical activity from adolescence to adulthood (31). Physical activity levels are reported to peak in early to mid-adolescence and then decline, with a 10–20% reduction in the proportion of moderately to vigorously active adults between 20 and 40 years of age (32). Considering that these high levels of physical inactivity may continue to rise and contribute to worsening NCD risk patterns into adulthood, the challenge is not only to promote physical activity among adolescents but to ensure its sustainability in adulthood.

Environmental factors that likely prevent South Africans from participating in optimal physical activity include a lack of safety and high crime rates, and a lack of green areas and recreation facilities (33). Furthermore, better infrastructure in urban centres, with easier access to public transport, and the lower likelihood to walk great distances to access schools, as well as urbanisation providing easier access to sedentary activities, such as television, and so on, probably contributed to the higher prevalence of physical inactivity in urban compared to rural youth (34, 35).

The higher prevalence of physical inactivity in females may contribute to the higher prevalence of overweight/obesity and raised WHR compared to males. Other reasons for the gender differences in overweight/obesity may be that energy needs differ for boys and girls, as does the timing of sexual maturation (28). Furthermore, the view that overweight in African women is traditionally desirable and indicative of success and happiness may also contribute to the high prevalence of overweight/obesity (36). Cognisance must be taken of these socio-cultural issues as they may hinder the implementation of any effective intervention aimed at preventing NCDs. Innovative strategies are urgently needed to curb the development of overweight/obesity, particularly in young females, as recent data among South African scholars have shown that the prevalence of overweight/obesity are not only high but also on the rise (37).

Given that overweight/obesity and physical inactivity predominated in females while tobacco and alcohol use were highly prevalent among males, these differential gender patterns in NCD risk factors indicate that the future disease burden will show marked gender differences. Women will be more susceptible to metabolic and hypertension-related conditions while men will be predisposed to smoking- and alcohol-related diseases in accord with the premature mortality burden for South Africa in 2000 (38).

Limitations of the study, not accounting for the socio-demographic characteristics, such as education, occupation, household size, marital status, and so on, include that, while similar questions were used in both surveys to ascertain smoking and alcohol history, these were self-reported. Another limitation is that the nutrition indicators in the SADHS were newly introduced in 2003 and need to be interpreted cautiously as these have not been validated extensively.

## Conclusion

The prevalences of modifiable NCD risk factors were high and well established in the South African black youth. Furthermore, the higher prevalence of certain risk factors in urban compared to rural youth suggests that unhealthy lifestyles may be expected to increase with further urbanisation. Considering that such high levels of risk factors have the potential to produce an epidemic of NCDs in the future, this is a matter of public health concern. Preventive strategies espousing a life-course approach are essential to curb the development of NCD risk factors (3). It is imperative that programmes target adolescents as it is during this vulnerable period that the long-term uptake of risky lifestyle behaviours occurs. While policies and guidelines related to the control of NCD risk factors have been developed in South Africa, translating them into effective programmes remains a challenging priority (18) and requires on-going surveillance.

## Appendix

**Table 1:** Selected chronic non-communicable disease risk factors in 15- to 24-year-old black youth presented by sex, age and year

Age (years)	Male						Female					
	
	15–16	17–18	19–20	21–22	23–24	Total	15–16	17–18	19–20	21–22	23–24	Total
1998												
Lifestyle												
Number	341	382	281	249	227	1481	343	333	331	356	342	1705
Smoke daily/occasionally, %	3.7	14.1	23.2	35.7	43.7	21.6	0.1	0.9	0.8	1.1	2.0	1.0
Problem drinking: CAGE ≥2, %	5.6	11.9	19.2	22.7	35.1	17.2	1.4	3.9	3.6	4.8	7.2	4.2
Anthropometry												
Number	331	376	275	241	220	1443	331	315	307	339	329	1620
BMI: mean (SE), kg/m^2^	19.8 (0.2)	20.5 (0.2)	21.0 (0.2)	21.5 (0.2)	21.7 (0.3)	20.8 (0.1)	22.0 (0.2)	23.1 (0.2)	23.5 (0.3)	24.7 (0.3)	24.6 (0.4)	23.6 (0.1)
BMI≥ 25 kg/m^2,^%	6.7	6.9	7.8	13.1	15.4	9.8	18.3	23.5	31.0	37.8	39.1	29.9
Waist circumference: mean (SE), cm	69.7 ( 0.4)	72.4 ( 0.4)	73.8 ( 0.5)	74.5 ( 0.5)	75.9 ( 0.6)	72.9 (0.2)	71.0 ( 0.5)	73.6 ( 0.5)	74.6 ( 0.6)	77.1 ( 0.6)	78.1 ( 0.8)	74.9 (0.3)
Waist-to-hip ratio (WHR): mean (SE)	0.81 (0.005)	0.78 (0.008)	0.83 (0.007)	0.78 (0.007)	0.82 (0.007)	0.8 (0.002)	0.81 (0.005)	0.82 (0.005)	0.82 (0.004)	0.78 (0.005)	0.76 (0.004)	0.8 (0.003)
Raised WHR, %	2.2	2.1	2.5	2.4	4.3	2.5	10.9	14.7	15.2	13.9	18.3	14.5
2003												
Lifestyle												
Number	233	229	208	174	138	983	212	245	233	203	189	1083
Smoke daily/occasionally, %	5.4	16.2	23.3	27.1	30.3	19.1	0.8	0.4	4.6	4.4	0.3	2.1
Problem drinking: CAGE ≥2, %	6.6	10.3	19.1	23.7	21.3	15.2	4.4	5.0	6.6	6.9	5.8	5.8
Physical activity < 600 METS/week	29.9	31.1	20.9	41.2	37.5	31.3	42.9	44.1	46.2	49.9	50.7	46.6
Nutrition												
Number	233	229	208	174	138	983	212	245	233	203	189	1083
Micronutrient intake: mean (SE)	50.2 (1.9)	48.9 (1.6)	45.5 (2.3)	45.2 (2.4)	40.9 (2.5)	46.7 (1.2)	46.8 (2.1)	45.7 (1.9)	50.1 (1.6)	43.7 (2.1)	45.1 (2.1)	46.4 (1.1)
Low micronutrient intake, %	20.3	19.4	16.5	16.3	8.7	16.9	17.2	16.0	15.8	14.9	15.2	15.9
High fat intake, % (3.5 + )	8.9	12.8	10.1	11.6	10.2	10.6	9.2	11.9	11.6	15.4	10.0	11.5
High salt intake, % (6 + )	10.6	16.4	11.4	14.5	12.2	12.9	15.8	8.4	10.4	10.7	8.7	10.9
Anthropometry												
Number	212	211	187	162	122	894	190	221	207	178	163	959
BMI: mean (SE), kg/m^2^	19.8 (0.3)	20.4 (0.2)	21.6 (0.3)	21.7 (0.3)	22.3 (0.5)	21 (0.1)	22.0 (0.3)	23.1 (0.3)	23.7 (0.4)	24.2 (0.5)	25.1 (0.5)	23.5 (0.2)
BMI≥ 25 kg/m^2,^%	8.3	6.6	11.4	13.4	14.1	10.1	17.9	21.4	32.8	39.7	44.1	31.1
Waist circumference: mean (SE), cm	68.4 ( 0.6)	68.7 ( 0.5)	73.3 ( 0.8)	72.8 ( 0.8)	73.6 ( 0.9)	71 (0.3)	69.1 ( 0.9)	72.2 ( 0.7)	74.7 ( 0.9)	75.9 ( 1)	77.1 ( 1)	73.6 (0.5)
Waist-to-hip ratio (WHR): mean (SE)	0.76 (0.007)	0.77 (0.006)	0.77 (0.006)	0.80 (0.006)	0.83 (0.005)	0.8 (.0003)	0.82 (0.005)	0.77 (0.005)	0.77 (0.006)	0.76 (0.005)	0.81 (0.004)	0.8 (0.004)
Raised WHR, %	1.4	0.5	0.6	1.3	1.0	1.0	9.1	16.5	22.7	18.0	17.0	13.1
